# A Study of the *Phaonia angelicae* Group (Diptera: Muscidae), with Descriptions of Six New Species from China

**DOI:** 10.1673/031.013.12901

**Published:** 2013-11-21

**Authors:** Wan-Qi Xue, Xiang Zhang

**Affiliations:** Institute of Entomology, Shenyang Normal University, Huanghe North Street 253, Huanggu District, Shenyang, 110034, China.

## Abstract

The *P. angelicae* group from China was studied, and six new species are described: *P. hanmiensis*, n. sp., *P. nanlingensis*, n. sp., *P. reduncicauda*, n. sp., *P. spargocerca*, n. sp., *P. subincana*, n. sp., and *P. varimargina*, n. sp. A key to the identification of males of the 40 Chinese species is given.

## Abstract (tr)

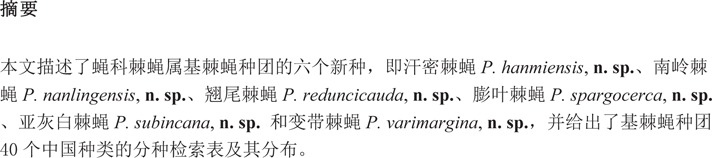

## Introduction

Robineau-Desvoidy (1830) established *Phaonia* (Diptera: Muscidae), with *P. viarum* as the type species. The genus and species from the Palaearctic Region were first divided into 16 groups (Ringdahl 1949), then 18 groups (Henning 1963), and later 21 groups and 3 subgroups (Zinovjev 1981). Following the work of these earlier researchers, Chinese scholars have concentrated on the study of *Phaonia*. Ma et al. (2002) divided the *Phaonia* species from China into 38 groups and 18 subgroups. The *P. angelicae* group was first presented by Ringdahl (1949) and divided into three subgroups by Zinovjev (1981). Now the *P. angelicae* group only includes the *P*. *angelicae* subgroup and the *P. hybrida* subgroup because Xue (2006) revised the *P. consobrina* subgroup because it has cerci that are deeply concave in the middle. In this paper, 40 Chinese species of the *P. angelicae* group are included, including six new species.

## Materials and Methods

The type specimens of six new species are all deposited at the Institute of Entomology, Shenyang Normal University, Shenyang, China.

The morphological terminology follows that of McAlpine (1981). Absolute measurements are used for the body length in millimeters (mm). The following abbreviations are used for characters: ***ors***, orbital setae; ***fr***, frontal setae; ***prst acr***, presutural acrostichal setae; ***acr***, acrostichal setae; ***prst dc***, presutural dorsocentral setae; ***dc***, dorsocentral setae; ***post dc***, postsutural dorsocentral setae; ***ial***, intraalar setae; ***pra***, prealar setae; ***av***, anteroventral setae; ***a***, anterior seta; ***ad***, anterodorsal setae; ***pd***, posterodorsal setae; ***p***, posterior setae; ***pv***, posteroventral setae; ***v***, ventral setae; **r-m**, radio-medial cross-vein; **dm-cu**, medio-cubital cross-vein; **R_4+5_**, branch of radius; and **M**, medial vein. Other abbreviations used are: **collect**., collector; **loc**., locality; and **IESNU**, Institute of Entomology, Shenyang Normal University, China.

***P. angelicae* group**
Diagnosis. **--** Epistoma situated in front of the anterior margin of frons or at the same vertical line in profile; thorax without presutural *acr*; basisternum of prosternum bare; mid tibia with *p* and *pv*; abdomen black; distal part of cerci with inboard processus and outboard processus.
Distribution. -- The 40 Chinese species of *P. angelicae* group are mainly distributed in Sichuan Province, China.
Bionomics. -- Relevant records are lacking.

**Key to species of *P. angelicae* group from China (♂♂)**
1. Fore tibia dense with long, brush-like hairs on the ventral surface2
— Fore tibia without above mentioned hairs on the ventral surface7
2. Prementum about 6.0 times as long as Broad3
— Prementum about 4.0 times as long as Broad4
3. Basal part of wing and haltere blackish brown; hind femur without *pv**P. kunjirapensis* Xue *et* Zhang, 1996
— Basal part of wing light brown and haltere yellow; hind femur with short *pv* in basal half*P. paederocerca* Feng *et* Ma, 2002
4. Mid tibia with 1–2 *ad*; hind femur without *pv*; outboard processus of cercus broad and big*P. biastostyla* Xue, 1998
— Mid tibia without *ad*5
5. Scape yellow, pedicel black, postpedicel blackish brown*P. subhybrida* Feng *et* Ma, 2002
— Antenna black6
6. Gena about 1/3 of eye in height; hind tibia with 1 sub-basal *pd**P. labidocerca* Feng *et* Ma, 2002
— Gena about 1/2 of eye in height; hind tibia without additional *pd**P. mengi* Feng, 2000
7. Scutum with 3 *post dc**P. longirostris* Xue *et* Zhao, 1998
— Scutum with 4 *post dc*8
8. Hind tibia without additional *pd*9
— Hind tibia with 1–3 short and weak *pd* in sub-basal24
9. Tibiae yellow or reddish brown, sometimes basal parts black10
— Tibiae black, sometimes basal parts reddish Brown12
10. Parafacial slightly wider than the postpedicel; the anterior margin of gena with 2 rows of upcurved subvibrissal setulae; mid tibia with 1 *pv*; each tergite with median black vita*P. sunwuensis* Xue *et* Ma, 1998
— Parafacial narrower than the postpedicel; the anterior margin of gena without above mentioned setulae; mid tibia with 2–3 *pv*; each tergite without median black vita11
11. Frons with *fr* row, extending to both sides of anterior ocellus, lower 2/5 strong and long; tibiae yellow; hind tibia with 3 *av*, 2 *ad**P. subemarginata* Fang, Li *et* Deng, 1986
— Frons only with 5 pairs of *fr* in lower half; tibiae black in basal, yellow in apical; hind tibia with 2 *av*, 1 *ad**P. daxiongi* Feng, 2001
12. Mid tibia with 1–2 *ad*13
— Mid tibia without *ad*15
13. Katepimeron bare*P. incana* (Wiedemann, 1817)
— Katepimeron with hairs14
14. Frons broad, about 2.0 times as wide as the distance between outer margins of posterior ocelli; fore tibia with 1 median *p*; each tergite with median black vitta, covered with grayish yellow pruinosity*P. zhangyeensis* Ma *et* Wu, 1992
— Frons narrow, subequal in width to anterior ocellus; fore tibia with 2 median *p*; each tergite with unconspicuous median black vitta, densely covered with gray pruinosity*P. subconsobrina* Ma, 1992
15. Epistoma snout-shaped, distinctly situated in front of the anterior margin of frons in profile16
— Epistoma not snout-shaped, slightly situated front of the anterior margin of frons, or at the same vertical line in profile 18
16. Frons broad, subequal to the distance between outer margins of posterior ocelli; fore tibia with 1 median *p**P. scrofigena* Ma *et* Xue, 1998
— Frons narrow, about 1.5 times as wide as anterior ocellus; fore tibia with 2 median *p*17
17. Haustellum about 2.5 times as long as palpus; mid femur only with *pv* in basal half; mid tibia with 1 *pd*, 3 *pv*; hind femur completely with *pv* row; hind tibia with 5 *ad**P. latimargina* Fang *et* Fan, 1988
— Haustellum about 1.5 times as long as palpus; mid femur with *pv* row, basal 2/3 strong and long; mid tibia with 3–4 *pd*, 1 *pv*; hind femur without *pv* in basal half; hind tibia with 3 *ad**P. varimargina* Xue *et* Zhang, n. sp.
18. Fore tibia without median *p*19
— Fore tibia with median 1–2 *p*20
19. Hind tibia with 2 rows of hair-like *ad*, 2 rows of short and weak *pv* in the middle; thorax and abdomen aeneous in ground color, covered with grayish white pruinosity*P. curvicercalis* Wei, 1990
— Hind tibia with 3 *ad*, without *pv* in the middle; thorax and abdomen black in ground color, sparsely covered with pruinosity*P. papillaria* Fang *et* Fan, 1993
20. Basicosta brownish yellow; surfaces of upper ommatidia distinctly enlarged; gena about 1/3 of eye in height; hind femur with long and strong *av* in apical half; hind tibia with 2–4 *ad*, without *p**P. planeta* Feng *et* Ma, 2002
— Basicosta black21
21. Gena about 1/2 of eye in height*P. megistogenysa* Feng *et* Ma, 2002
— Gena about 1/5–1/4 of eye in height22
22. Parafacial about 2.0 times as wide as the postpedicel; the anterior margin of gena with 2 rows of upcurved subvibrissal setulae; *pra* subequal in length to posterior notopleural seta; apical parts of femora, and basal parts of tibiae reddish brown; hind tibia with a row of short *p* in the middle*P. zhougongshana* Ma *et* Feng, 2002
— Parafacial subequal in width to the postpedicel; the anterior margin of gena with a row of upcurved subvibrissal setulae at most; *pra* about 1.5 times as long as posterior notopleural seta at least; legs all black, hind tibia without *p*23
23. Facial carina projecting, nearly buninoid; katepimeron with hairs; hind tibia only with 2 *ad*; cersus with inconspicuous inboard processus*P. hanmiensis* Xue *et* Zhang, n. sp.
— Facial carina not projecting; katepimeron bare; hind tibia with a row of *ad*; cersus with distinct inboard processus*P. nanlingensis* Xue *et* Zhang, n. sp.
24. Tibiae reddish brown or yellow in apical Half25
— Legs all black or brownish black, sometimes apical parts of femora, and basal parts of tibiae yellow26
25. Gena about 1/3 of eye in height; the anterior margin of gena with a row of upcurved subvibrissal setulae; hind femur with 3–4 *pv* in basal thirds*P. fuscitibia* Shinonaga *et* Kano, 1971
— Gena about 1/2 of eye in height; the anterior margin of gena with 2 rows of upcurved subvibrissal setulae; hind femur without *pv**P. nigeritegula* Feng, 2002
26. Katepimeron bare27
— Katepimeron with hairs30
27. Apical parts of femora, and basal parts of tibiae yellow*P. wulinga* Xue, 1998
— Legs all black28
28. Epistoma not projecting, at the same vertical line with the anterior margin of frons in profile*P. subincana* Xue *et* Zhang, n. sp.
— Epistoma snout-shaped, distinctly situated front of the anterior margin of frons in profile29
29. Each tergite without shining patches; dmcu not clouded*P. mimoincana* Ma *et* Feng, 1986
— Tergite 5 with a pair of shining lateral patches; dm-cu clouded*P. reduncicauda* Xue *et* Zhang, n. sp.
30. Mid tibia with *ad*31
— Mid tibia without *ad*33
31. Fronto-orbital plate, parafacial and gena covered with golden pruinosity; mid tibia with 3–4 *ad**P. hamiloba* Ma, 1992
— Fronto-orbital plate, parafacial and gena covered with gray pruinosity; mid tibia with 1 *ad*32
32. Wing hyaline; hind femur with *pv* row in apical; abdomen long oviform, each tergite with median black vitta, without shining patches*P. hunyuanensis* Ma *et* Wang, 1998
— Wing light brown; hind femur without *pv*; abdomen short cone-shaped, with shifting pathes, without median vittae*P. maowenensis* Deng *et* Feng, 1998
33. Fore tibia without median *p*; abdomen without median vittae and shining patches*P. ningxiaensis* Ma *et* Zhao, 1992
— Fore tibia with median *p*34
34. Scape and pedicel all dark brown, or scape Yellow35
— Antenna black36
35. Scape and pedicel dark brown; gena about 1/3 of eye in height; the anterior margin of gena with 2 rows of upcurved subvibrissal setulae; tergite 3 and 4 with median black vita*P. fusciantenna* Feng *et* Ma, 2002
— Scape yellow; gena about 1/2 of eye in height; the anterior margin of gena with 3 rows of upcurved subvibrissal setulae; each tergite with median black vita*P. megacerca* Feng *et* Ma, 2002
36. Parafacial with a white patch in upper half; prementum about 7.0 times as long as broad; scutum with 3 black vittae*P. longipalpis* Feng *et* Ma, 2002
— Parafacial without patches; prementum about 4.0 times as long as broad at most; scutum with 4 black vittae, or without vittae37
37. Scutum with 3 *prst dc*; gena reddish brown in upper half*P. jiagedaqiensis* Ma *et* Cui, 1992
— Scutum with 2 *prst dc*; gena black38
38. *pra* slightly longer than or subequal to posterior notopleural seta; each tergite without median black vita*P. spargocerca* Xue *et* Zhang, n. sp.
— *pra* long and strong, about 2.0 times as long as posterior notopleural seta; tergite 3 and 4 with median black vittae39
39. Frons about 2.0 times as wide as anterior ocellus; gena about 1/2 of eye in height; the anterior margin of gena with 2 rows of upcurved subvibrissal setulae; apical parts of femora, and basal parts of tibiae reddish brown*P. wanfodinga* Feng *et* Ma, 2002
— Frons subequal in width to anterior ocellus; gena about 1/4 of the eye in height, the anterior margin of gena without upcurved subvibrissal setulae; legs all black*P. paomashanica* Feng, 2004

**Figure 1.**
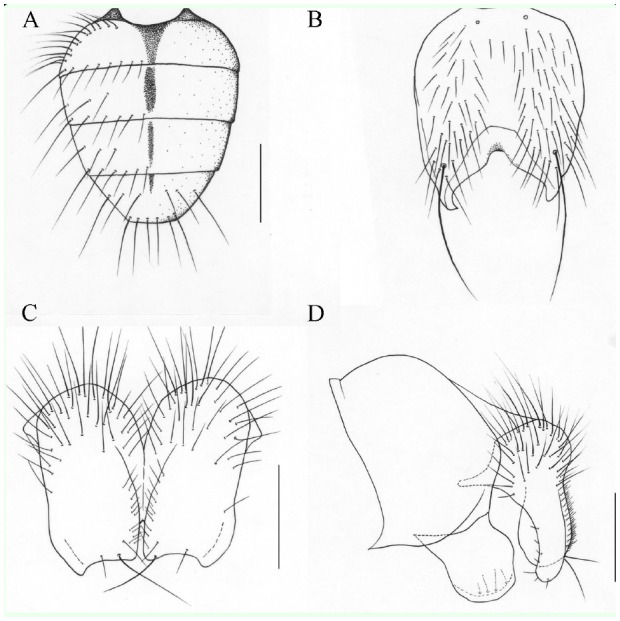
*Phaonia hanmiensis*, n. sp. (A) male, abdomen in dorsal view; (B) male, sternite 5 in ventral view; (C) male, cerci in posterior view; (D) male, cersus and surstylus in profile. Scale bars: A, 1.0 mm; B, 0.5 mm; C and D, 0.2 mm. High quality figures are available online.

**Figure 2.**
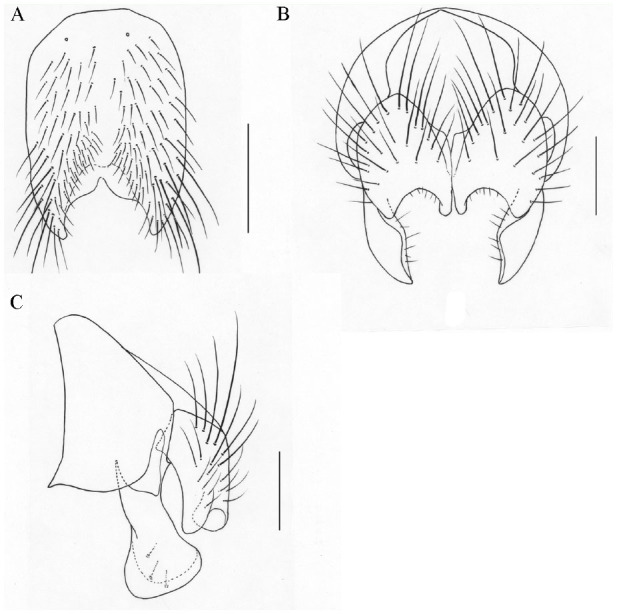
*Phaonia nanlingensis*, n. sp. (A) male, sternite 5 in ventral view; (B) male, cerci and surstyli in posterior view; (C) male, cersus and surstylus in profile. Scale bars: A, 0.5 mm; B and C, 0.2 mm. High quality figures are available online.

**Figure 3.**
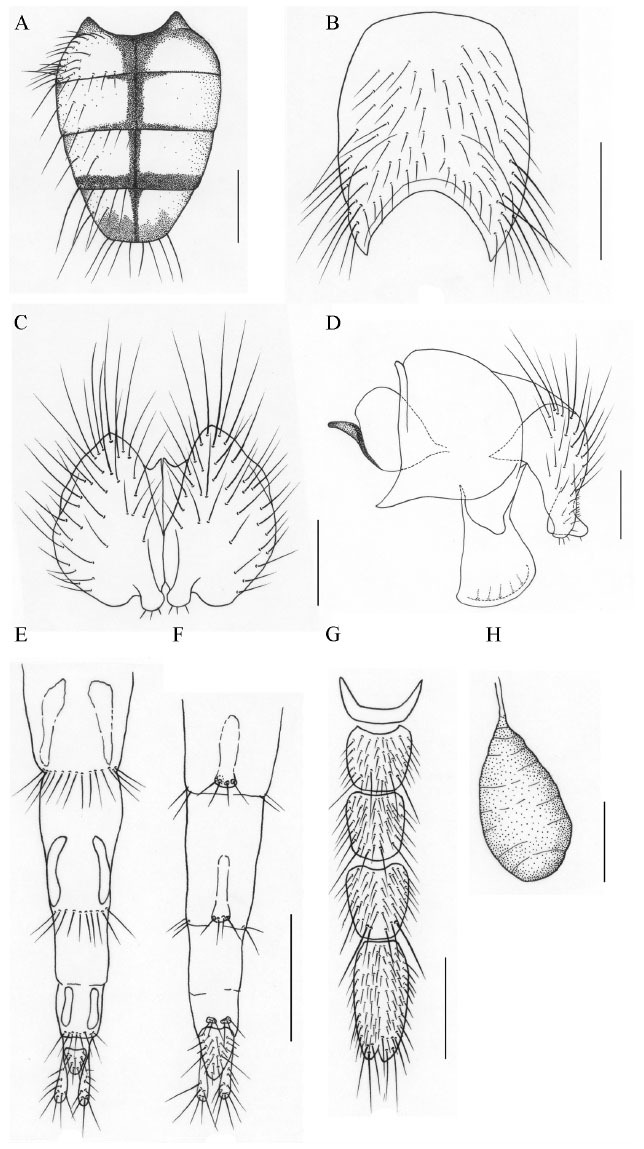
*Phaonia reduncicauda*, n. sp. (A) male, abdomen in dorsal view; (B) male, sternite 5 in ventral view; (C) male, cerci in posterior view; (D) male, cersus and surstylus in profile; (E) female, ovipositor in dorsal view; (F) female, ovipositor in ventral view; (G) female, sternites 1–5; (H) female, spermatheca. Scale bars: A, E, F, and G, 1.0 mm; B, 0.5 mm; C and D, 0.2 mm; H, 0.1 mm. High quality figures are available online.

**Figure 4.**
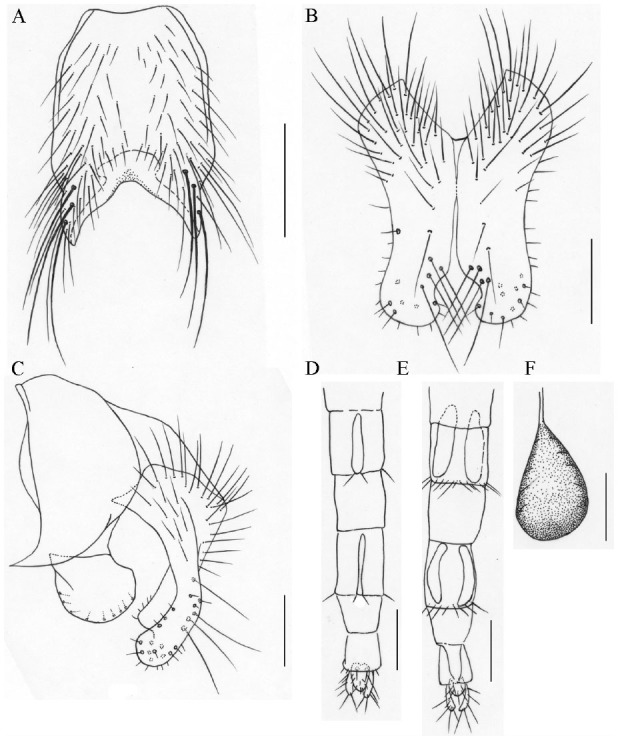
*Phaonia spargocerca*, n. sp. (A) male, sternite 5 in ventral view; (B) male, cerci in posterior view; (C) male, cersus and surstylus in profile; (D) female, ovipositor in ventral view; (E) female, ovipositor in dorsal view; (F) female, spermatheca. Scale bars: A, 0.5 mm; B and C, 0.2 mm; D and E, 1.0 mm; F, 0.1 mm. High quality figures are available online.

**Figure 5.**
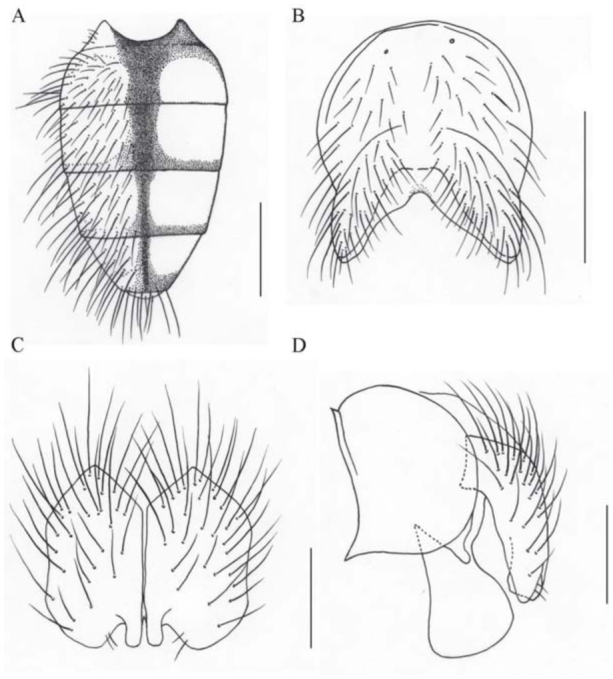
*Phaonia subincana*, n. sp. (A) male, abdomen in dorsal view; (B) male, sternite 5 in ventral view; (C) male, cerci in posterior view; (D) male, cersus and surstylus in profile. Scale bars: A, 1.0 mm; B, 0.5 mm; C and D, 0.2 mm. High quality figures are available online.

**Figure 6.**
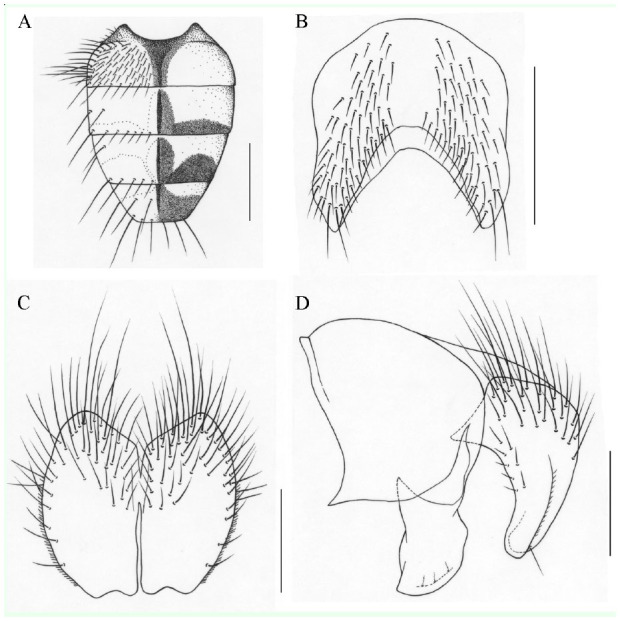
*Phaonia varimargina*, n. sp. (A) male, abdomen in dorsal view; (B) male, sternite 5 in ventral view; (C) male, cerci in posterior view; (D) male, cersus and surstylus in profile. Scale bars: A, 1.0 mm; B, 0.5 mm; C and D, 0.2 mm. High quality figures are available online.

**1. *P. biastostyla*Xue, 1998: 1222**
  **Distribution. --** China: Tonghua City (type loc.), Jilin Province; Benxi City, Liaoning Province.
**2. *P. curvicercalis*Wei, 1990: 497**
  **Distribution. --** China: Pan County (type loc.), Guizhou Province.
**3. *P. daxiongi*Feng, 2001: 30**
  **Distribution. --** China: Mt. Erlang (type loc.), Yaan City, Sichuan Province.
**4. *P. fusciantenna*Feng *et* Ma, 2002: 94**
  **Distribution. --** China: Mt. Zhougong (type loc.), Yaan City, Sichuan Province.
**5. *P. fuscitibia*Shinonaga *et* Kano, 1971: 177**
  **Distribution. --** Japan: Sapporo (type loc.). China: Jiagedaqi, Heilongjiang Province; Mt. Changbai, Jilin Province. Mongolia. Russia.
**6. *P. hamiloba* Ma, 1992: 443**
  **Distribution. --** China: Yushu County (type loc.), Qinghai Province; Kangding County, Sichuan Province; Mt. Houding, Zhangjiakou City, Hebei Province.
**7. *P. hanmiensis* Xue *et* Zhang, n. sp. (Figure 1 A–D)**
  **Holotype male. --** Body length 6.0–6.2 mm.
  ***Head***. Eye covered with long ciliae; frons subequal in width to anterior ocellus, frontal vitta black, disappearing in the middle; 10 pairs of *fr*, extending to both sides of anterior ocellus, upper 4–5 short, which are subequal in length to eye ciliae, without *ors*; frontoorbital plate, parafacial and gena covered with pale gray pruinosity, parafacial subequal in width to the postpedicel; antenna black, postpedicel about 2.5 times as long as broad; arista long plumose, the longest hair about 2.0 times as long as the width of the postpedicel; facial carina projecting, nearly buninoid; vibrissal angle situated in front of frontal angle in profile; gena about 1/5 of eye in height, the anterior margin of gena with a row of upcurved subvibrissal setulae, genal and postgenal hairs black; prementum about 2.5 times as long as broad, covered with gray pruinosity and hairs; palpus black, longer than prementum.
  ***Thorax***. Black in ground color, sparsely covered with gray pruinosity; scutum with 4 black vittae, scutellum black; 8–10 rows of hair-like *prst acr*, *acr* 0+1, *dc* 2+4, *ial* 0+2, *pra* long and strong, about 1.5 times as long as posterior notopleural seta; notopleuron and katepimeron with hairs, lateral and ventral surface of scutellum, basisternum of prosternum, and meron all bare; spiracles dark brown; katepisternal setae 1+2.
  ***Wing***. Light brown, veins dark brown, basicosta black; costal spines absent, dorsal and ventral surface of radial node all bare; distal parts of R4+5 and M straight, r-m and dm-cu not clouded; calypters and haltere brownyellow.
  ***Legs***. All black; fore tibia with 2 median *p*, without long hairs on the ventral surface; mid femur with a row of short and weak *av*, a row of *pv* (3 of them long and strong in the basal half); mid tibia with 2 *p*, 2 *pv*, without *ad*; hind femur with *av* in the apical half, without *pv*; hind tibia with 3–4 *av*, 2 *ad*, 1 *pd* in apical quarter, without additional sub-basal *pd* and apical *pv*; all tarsi longer than tibiae; claws and pulvilli short and small.
  ***Abdomen***. Black in ground color, nearly rounded in dorsal view, covered with gray pruinosity; tergites 4 and 5 slightly shiny, each tergite with median narrow black vitta, without shining patches; sternite 1 bare, the posterior part of every sternite 2–5 with a pair of long setae; cercus with many thin hairs on inner margin, with inconspicuous inboard processus.
  **Female. --** Unknown.
  **Type material. -- *Holotype***. China, Tibet, Motuo County, Hanmi, 2150-3200 m a.s.l., 9 August 2003, Mingfu Wang Collect., ♂ (IESNU). ***Paratype***. Same data as holotype. ♂ (IESNU).
  **Remarks. --** This new species is similar to *P. zhougongshana* Ma *et* Feng, 2002, but it differs from the latter in the male: parafacial subequal in width to the postpedicel; anterior margin of gena with a row of upcurved subvibrissal setulae; *pra* long and strong, about 1.5 times as long as posterior notopleural seta; katepimeron with hairs; legs all black; hind tibia with 2 *ad*; cersus with many thin hairs on inner margin, without distinct inboard processus.
  **Etymology.** — The species name is based on the place of collection, Hanmi.
  **Distribution. --** China: Tibet.
**8. *P. hunyuanensis* Ma *et* Wang, 1998: 1221**
  **Distribution. --** China: Hunyuan County (type loc.), Shanxi Province.
**9. *P. incana* (Wiedemann, 1817): 81**
  **Distribution. --** Germany: Kiel (type loc.). China: Mt. Changbai, Jilin Province; Haiyan County, Maqin County, Qinghai Province. Mongolia. Russia.
**10. *P. jiagedaqiensis* Ma *et* Cui, 1992: 924**
  **Distribution. --** China: Jiagedaqi (type loc.), Heilongjiang Province.
**11. *P. kunjirapensis*Xue *et* Zhang, 1996: 225**
  **Distribution. --** China: Kunjirap (type loc.), Tashikuergantajike Autonomous County, Xinjiang Uygur Autonomous Region.
**12. *P. labidocerca*Feng *et* Ma, 2002: 58**
  **Distribution. --** China: Mt. Erlang (type loc.), Yaan City, Sichuan Province.
**13. *P. latimargina*Fang *et* Fan, 1988: 503**
  **Distribution. --** China: Mt. Galongla (type loc.), Motuo County, Tibet.
**14. *P. longipalpis*Feng *et* Ma, 2002: 96**
  **Distribution. --** China: Mt. Erlang (type loc.), Yaan City, Sichuan Province.
**15. *P. longirostris*Xue *et* Zhao, 1998: 1233**
  **Distribution. --** China: Mt. Xiaowutai (type loc.), Hebei Province.
**16. *P. maowenensis*Deng *et* Feng, 1998: 95**
  **Distribution. --** China: Sanlong (type loc.), Maowen County, Sichuan Province.
**17. *P. megacerca*Feng *et* Ma, 2002: 95**
  **Distribution. --** China: Mt. Erlang (type loc.), Yaan City, Sichuan Province.
**18. *P. megistogenysa*Feng *et* Ma, 2002: 86**
  **Distribution. --** China: Mt. Erlang (type loc.), Yaan City, Sichuan Province.
**19. *P. mengi*Feng, 2000: 203**
  **Distribution. --** China: Mt. Jiaoding (type loc.), Hanyuan County, Sichuan Province.
**20. *P. mimoincana*Ma *et* Feng, 1986: 89**
  **Distribution. --** China: Mt. Erlang (type loc.), Yaan City, Sichuan Province.
**21. *P. nanlingensis* Xue *et* Zhang, n. sp. (Figure 2 A–C)**
  **Holotype male. --** Body length 7.0–7.2 mm.
  ***Head***. Eye sparsely covered with long ciliae, surfaces of inboard upper ommatidia not enlarged; frons subequal in width to anterior ocellus, frontal vitta black, disappearing in the middle; 12–14 pairs of *fr*, extending to both sides of anterior ocellus, upper half short and subequal in length to eye ciliae, without *ors*; fronto-orbital plate, parafacial and gena covered with pale gray pruinosity, parafacial subequal in width to the postpedicel; antenna black, postpedicel about 3.0 times as long as broad; arista long plumose, the longest hair about 1.5 times as long as the width of the postpedicel; vibrissal angle slightly situated in front of frontal angle in profile; gena about 1/4 of the eye in height, genal and postgenal hairs black; proboscis short, prementum about 3.0 times as long as broad, covered with gray pruinosity and hairs; palpus black, longer than prementum.
  ***Thorax***. Black in ground color, sparsely covered with caesious pruinosity; scutum with 4 black vittae, scutellum black; *acr* 0+1, *dc* 2+4, *ial* 0+2, *pra* long and strong, about 2.0 times as long as posterior notopleural seta; notopleuron with hairs; lateral and ventral surface of scutellum, basisternum of prosternum, katepimeron, and meron all bare; spiracles dark brown; katepisternal setae 1+2.
  ***Wing***. Light brown, veins dark brown, basicosta black, subcostal sclerite light brown; costal spines short and weak; dorsal and ventral surfaces of radial node all bare; distal parts of R4+5 and M straight; r-m and dm-cu not clouded; calypters light brown, haltere yellow.
  ***Legs***. All black; fore tibia with 1 median *p*, without long hairs on ventral surface; mid femur with 3–4 *pv* in basal half, without *av* row; mid tibia with 2–3 *p*, 2 *pv*, without *ad*; hind femur with *av* row, without *pv*; hind tibia with 4 *av*, a row of *ad*, 1 *pd* in apical fifth, without apical *pv*; all tarsi longer than tibiae; claws and pulvilli long, subequal in length to fourth tarsomere.
  ***Abdomen***. Black in ground color, nearly rounded in dorsal view, sparsely covered with caesious pruinosity; each tergite with median black vitta, without shining patches; sternite 1 bare; cersus with distinct inboard processus; surstylus slender, but distal apart enlarged.
  **Female. --** Unknown.
  **Type material. -- *Holotype***. China, Guangdong Province, Shaoguan City, Nanling National Park, Mt. Xiaohuang, 850–1900 m a.s.l., 22 July 2004, Chuntian Zhang Collect., *♂* (IESNU). ***Paratype***. Same data as holotype. 5 *♂♂* (IESNU).
  **Remarks. --** This new species is similar to *P. zhougongshana* Ma *et* Feng, 2002, but it differs from the latter in the male: eye sparsely covered with long ciliae; parafacial subequal in width to the postpedicel; legs all black; hind tibia with 4 *av*, without *a*, *p*; surstylus slender, but distal part enlarged.
  **Etymology.** — The species name is based on the place of collection, Nanling, where Mount Xiaohuangshan lies.
  **Distribution. --** China: Guangdong Province.
**22. *P. nigeritegula*Feng, 2002: 3**
  **Distribution. --** China: Taiziping (type loc.), Mt. Emei, Sichuan Province.
**23. *P. ningxiaensis* Ma *et* Zhao, 1992: 924**
  **Distribution. --** China: Xixia (type loc.), Jingyuan County, Ningxia Hui Autonomous Region.
**24. *P. paederocerca*Feng *et* Ma, 2002: 55**
  **Distribution. --** China: Wanfoding (type loc.), Mt. Emei, Sichuan Province; Mt. Jiaoding, Hanyuan County, Sichuan Province.
**25. *P. paomashanica*Feng, 2004: 8**
  **Distribution. --** China: Mt. Paoma (type loc.), Kangding County, Sichuan Province.
**26. *P. papillaria*Fang *et* Fan, 1993: 1241**
  **Distribution. --** China: Qingtiange (type loc.), Weixi Lisu Autonomous County, Yunnan Province.
**27. *P. planeta*Feng *et* Ma, 2002: 86**
  **Distribution. --** China: Mt. Erlang (type loc.), Yaan City, Sichuan Province.
**28. *P. reduncicauda* Xue *et* Zhang, n. sp. (Figure 3 A–H)**
  **Holotype male. --** Body length 7.8 mm.
  ***Head***. Eye covered with long dark brown ciliae; frons subequal in width to the distance between outer margins of posterior ocelli, frontal vitta black, subequal in width to fronto-orbital plate; 11 pairs of *fr*, extending to both sides of anterior ocellus, upper 5 short, which are subequal in length to eye ciliae, without *ors*; fronto-orbital plate, parafacial and gena covered with gray pruinosity, parafacial with a silvery white patch in the upper half; antenna black, postpedicel about 2.5 times as long as broad; arista plumose, the longest hair slightly longer than or subequal to the width of the postpedicel; facial carina projecting, relatively narrower; epistoma snoutshaped, distinctly situated front of the anterior margin of frons in profile; gena about 2/5 of eye in height, the anterior margin of gena with 2–3 rows of upcurved subvibrissal setulae, genal and postgenal hairs all black; prementum about 2.5 times as long as broad, covered with gray pruinosity and hairs; palpus black, longer than prementum.
  ***Thorax***. Black in ground color, densely covered with gray pruinosity; scutum with 4 black vittae, scutellum black; 6 rows of hair-like *prst acr*, *acr* 0+1, *dc* 2+4, *ial* 0+2, *pra* long and strong, about 1.5 times as long as posterior notopleural seta; notopleuron with hairs; lateral and ventral surface of scutellum, basisternum of prosternum, katepimeron, and meron all bare; spiracles dark brown; katepisternal setae 1+2.
  ***Wing***. Light brown, veins dark brown, basicosta black; costal spines short, about 1/2 of r-m in length; dorsal and ventral surface of radial node all bare; distal parts of R4+5 and M straight; dm-cu clouded; calypters and haltere brownish yellow.
  ***Legs***. All black; fore tibia with 2 median *p*, without long hairs on ventral surface; mid femur with *pv* row, which was stronger in the basal half, without *av* row; mid tibia with 3 *p*, 2 *pv*, without *ad*; hind femur with *av* in the apical half, without *pv*; hind tibia with 3–4 *av*, 4 *ad*, 1 *pd* in apical quarter, additional 1–2 *pd* in basal third, without apical *pv*; all tarsi longer than tibiae; claws and pulvilli short.
  ***Abdomen***. Black in ground color, oviform in dorsal view, densely covered with bluish gray pruinosity; each tergite with median black vitta, posterior margin of tergite 3 and 4 with stripes, tergite 5 with a pair of shining lateral patches, other tergites without shining patches; sternite 1 bare; distal part of inner processus of cersus enlarged, and curved backward; ejaculatory apodeme big globose.
  **Female. --** Body length 6.8–7.8 mm. Frons about 1/3 of head in width, frontal vitta about 4.0 times as wide as fronto-orbital plate 5 *fr*, 2 *ors*; frontal triangle reaching to the middle of frons; parafacial about 1.5 times as wide as the postpedicel; the longest aristal hair about 1.2 times as long as the width of the postpedicel; fore tibia with 1 median *p*; mid tibia with 1–2 *pv*; hind tibia with 2–3 *ad*; posterior margin of tergite 3 with relatively broader stripe; the other characters as male.
  **Type material. -- *Holotype***. China, Tibet, Motuo County, Mt. Duoxiongla, 3600–4200 m a.s.l., 8 August 2003, Mingfu Wang Collect., ♂ (IESNU). ***Paratype***. Same data as holotype. 2 ♀♀ (IESNU).
  **Remarks. --** This new species is similar to *P. mimoincana* Ma *et* Feng, 1986, but it differs from the latter in the male: frons subequal in width to the distance between outer margins of posterior ocelli; parafacial with a silvery white patch in the upper half; arista plumose, the longest hair slightly longer than or subequal to the width of the postpedicel; gena about 2/5 of eye in height; costal spines short, about 1/2 of r-m in length; dm-cu clouded; mid femur with *pv* row, which was stronger in the basal half; posterior margin of tergite 3 and 4 with stripes; tergite 5 with a pair of shining lateral patches.
  **Etymology. --** The species name is derived from the Latin words “*reduncus*” meaning curved backward and “*cauda*” meaning tail, referring to the inner processus of the cersus being curved backward.
  **Distribution. --**China: Tibet.
**29. *P. scrofigena* Ma *et*Xue, 1998: 1266**
  **Distribution. --** China: Barkam County (type loc.), Sichuan Province.
**30. *P. spargocerca* Xue *et* Zhang, n. sp. (Figure 4 A–F)**
  **Holotype male. --** Body length 8.0 mm.
  ***Head***. Eye densely covered with light brown ciliae; frons narrow, subequal in width to anterior ocellus, fronto-orbital plates adjoining in the middle; 13 pairs of *fr*, extending to both sides of anterior ocellus, upper 7 short, which were subequal in length to the eye ciliae, without *ors*; fronto-orbital plate, parafacial and gena covered with dark gray pruinosity, parafacial about 1.75 times as wide as the postpedicel; antenna black, postpedicel about 3.0 times as long as broad; arista long plumose, the longest hair about 1.5 times as long as width of the postpedicel; vibrissa angle distinctly situated in front of the frontal angle in profile; gena about 2/5 of eye in height, the anterior margin of gena with a row of upcurved subvibrissal setulae, genal and postgenal hairs all black; the upper lateral area of occiput with hairs; proboscis short, with a pair of prestomal teeth; prementum about 2.5 times as long as broad, covered with pruinosity; palpus black and slender, slightly longer than prementum.
  ***Thorax***. Black in ground color, scutum with 4 inconspicuous black-brown vittae, scutellum black; *acr* 0+1, *dc* 2+4, *ial* 0+2, *pra* slightly longer than or subequal to posterior notopleural seta; both sides of scutellum with few hairs, ventral surface bare; notopleuron and katepimeron with hairs, basisternum of prosternum, proepisternum, anepimeron, and meron all bare; anterior spiracle light yellow to white, posterior spiracle light brown; katepisternal setae 1+2.
  ***Wing***. Light brown, veins dark brown, basicosta black; costal spines short and weak; dorsal and ventral surface of radial node all bare; distal parts of R4+5 and M straight; r-m and dm-cu not clouded; calypters light brown, the lower calypter projecting; haltere brown yellow.
  ***Legs***. All black; fore tibia with 3 *pv* in the apical half, without long hairs on the ventral surface; mid femur with strong *av* row, 1 preapical *a*, 2 *pd*, 3–4 blunt-pointed *pv* in the basal half; mid tibia with 1 *p*, 2 *pv*, without *ad*; hind femur with a row of *av*, which become long and strong apically, without *pv*; hind tibia with 4 short and weak *av* in the middle, complete *ad* row, 1 long and strong *pd* in the apical quarter, 1 additional *pd* basal, without *p* and apical *pv*; all tarsi longer than tibiae; claws and pulvilli short.
  ***Abdomen***. Black in ground color, oviform in dorsal view, without vittae and shining patches; sternite 1 bare.
  **Female. --** Body length 9.0 mm. Frons about 3/8 of the head in width, fronto-orbital plate about 1/4 of the frontal vitta in width, frontal vitta black; frontal triangle covered with brownish yellow pruinosity, reaching to upper 2/5 of frons; 8 pairs of *fr*, 2 pairs of *ors*; fronto- orbital plate and upper parafacial covered with gray pruinosity, lower parafacial and gena covered with brownish yellow pruinosity; parafacial about 2.0 times as wide as the postpedicel; arista long plumose, the longest hair about 1.2 times as long as the width of the postpedicel; gena about 1/2 of the eye in height; prementum sparsely covered with pruinosity; scutum distinct with black brown vittae; both sides of scutellum with few hairs; dm-cu clouded; fore tibia with median *p*; mid tibia with 2 *ad*, 3–4 *p*, 2 *pv*; hind tibia with 2–3 *av*; hind part of tergite 1, fore part of tergites 3 and 4 densely covered with gray pruinosity; tergites 3 and 4 with median vittae; the other characters as male.
  **Type material. -- *Holotype***. China, Yunnan Province, Shangri-La County, Bita Lake, 4000–4150 m, 2 July 2002, Wanqi Xue Collect., *♂* (IESNU). ***Paratype***. Same data as holotype, ♀ (IESNU).
  **Remarks. --** This new species is similar to *P. wanfodinga* Feng *et* Ma, 2002, but it differs from the latter in the male: frons narrow, subequal in width to anterior ocellus; parafacial about 1.75 times as wide as the postpedicel; arista long plumose, the longest hair about 1.5 times as long as the width of the postpedicel; gena about 2/5 of the eye in height, the anterior margin of gena with a row of upcurved subvibrissal setulae; *pra* slightly longer than or subequal to posterior notopleural seta; anterior spiracle light yellow to white.
  **Etymology. --** The species name is derived from the Greek words “*spargosis*” meaning enlarged and “*cercus*” meaning cercus, referring to male cerci being broad in the apical part.
  **Distribution. --** China: Yunnan Province.
**31. *P. subconsobrina* Ma, 1992: 923**
  **Distribution. --** China: Mt. Changbai (type loc.), Jilin Province.
**32. *P. subemarginata*Fang, Li *et* Deng, 1986: 241**
  **Distribution. --** China: Mt. Emei (type loc.), Sichuan Province.
**33. *P. subhybrida*Feng *et* Ma, 2002: 58**
  **Distribution. --** China: Mt. Erlang (type loc.), Yaan City, Sichuan Province.
**34. *P. subincana* Xue *et* Zhang, n. sp. (Figure 5 A–D)**
  **Holotype male. --** Body length 6.0–6.2 mm.
  ***Head***. Eye sparsely covered with short light brown ciliae; frons about 1.2 times as wide as the distance between outer margins of posterior ocelli; frontal vitta black, the narrowest part about 2.0 times as wide as the fronto-orbital plate; 11 pairs of *fr*, extending to both sides of anterior ocellus, without *ors*; fronto-orbital plate, parafacial and gena sparsely covered with gray pruinosity, parafacial about 1.5 times as wide as the postpedicel; antenna black, postpedicel about 2.5 times as long as broad; arista ciliated, the longest hair slightly shorter than the width of half the postpedicel; epistoma at the same vertical line with the anterior margin of frons in profile; gena about 1/2 of the eye in height, the anterior margin of gena with 3 rows of upcurved subvibrissal setulae, genal and postgenal hairs all black; prementum about 2.5 times as long as broad, covered with gray pruinosity; palpus black and slender, slightly longer than the prementum.
  ***Thorax***. Black in ground color, sparsely covered with brownish gray pruinosity; scutum with 4 black vittae, scutellum black; 4 rows of hair-like *prst acr*, *acr* 0+1, *dc* 2+4, *ial* 0+2, *pra* about 2.0 times as long as posterior notopleural seta; notopleuron with hairs, lateral and ventral surface of scutellum, basisternum of prosternum, katepimeron, and meron all bare; spiracles dark brown; katepisternal setae 1+2.
  ***Wing***. Light brown, basal part of wing and veins dark brown, basicosta black; costal spines short and weak; dorsal and ventral surface of radial node all bare; subcosta bowlike, distal parts of R4+5 and M straight; r-m and dm-cu not clouded; calypters pale brown, haltere light brown in basal, black in apical.
  ***Legs***. All black; fore tibia with 1 median *p*, without long hairs on the ventral surface; mid femur with a row of short *av*, a row of *pv* that become short apically; mid tibia with 1 short and weak *ad*, 2–3 *p*, 1–2 *pv*; hind femur with *av* row, which is long and strong in the apical 2/5, without *pv*; hind tibia with 2–3 *av*, 4–5 *ad*, 1 long and strong *pd* in the apical third, 1 short and weak *pd* in the middle, 2 short and weak *pd* in the sub-basal region; all tarsi longer than tibiae; claws and pulvilli short.
  ***Abdomen***. Black in ground color, long oviform in dorsal view, covered with dark gray pruinosity and spindly setae; each tergite with median black vitta, but the median black vittae of tergites 4 and 5 narrower; posterior margins of tergites 3–5 with black stripes; sternite 1 bare; cerci nearly quadrate in posterior view, outboard processus broad, and longer than inboard processus.
  **Female. --** Unknown.
  **Type material. -- *Holotype***. China, Tibet, Motuo County, Mt. Duoxiongla, 3600–4200 m a.s.l., 8 August 2003, Mingfu Wang Collect., ♂ (IESNU). ***Paratype***. Same data as holotype. 2 ♂♂ (IESNU).
  **Remarks. --** This new species is similar to *P. mimoincana* Ma *et* Feng,1986, but it differs from the latter in the male: eye sparsely covered with short light brown ciliae; arista ciliated, the longest hair slightly shorter than the width of half of the postpedicel; gena about 1/2 of the eye in height; mid tibia with 1 short and weak *ad*; hind femur without *pv*; hind tibia with 1 short and weak *pd* in the middle; cerci nearly quadrate in the posterior view, outboard processus broad, and longer than the inboard processus.
  **Etymology. --** The species name is based on the new species being similar to *P. mimoincana* Ma *et* Feng, 1986.
  **Distribution. --** China: Tibet.
**35. *P. sunwuensis*Xue *et* Ma, 1998: 1281**
  **Distribution. --** China: Heilongjiang Province (type loc.).
**36. *P. varimargina* Xue *et* Zhang, n. sp. (Figure 6 A–D)**
  **Holotype male. --** Body length 6.0–6.2 mm.
  ***Head***. Eye covered with long ciliae, surfaces of upper ommatidia not enlarged; frons about 1.5 times as wide as the anterior ocellus, frontal vitta black, disappearing in the middle; 11 pairs of *fr*, extending to both sides of the anterior ocellus, upper 5 short and slightly longer than the eye ciliae, without *ors*; frontoorbital plate, parafacial and gena covered with gray pruinosity, parafacial about 1.5 times as wide as the postpedicel; antenna black, postpedicel about 2.5 times as long as broad; arista long plumose, the longest hair about 1.3 times as long as the width of the postpedicel; facial carina projecting, nearly buninoid; epistoma snout-shaped, distinctly situated in front of the anterior margin of frons in profile; gena about 2/5 of the eye in height, the anterior margin of gena with a row of upcurved subvibrissal setulae, genal and postgenal hairs all black; prementum without pruinosity; palpus black and slender; haustellum relatively shorter, about 1.5 times as long as the palpus.
  ***Thorax***. Black in ground color, sparsely covered with gray pruinosity; scutum with 4 black vittae, scutellum black; 8 rows of hair-like *prst acr*, *acr* 0+1, *dc* 2+4, *ial* 0+2, *pra* long and strong, about 2.0 times as long as the anterior notopleural seta, posterior notopleural seta short and weak, about 1/2 of the anterior notopleural seta in length; notopleuron with hairs; lateral and ventral surface of scutellum, basisternum of prosternum, katepimeron, and meron all bare; spiracles dark brown; katepisternal setae 1+2.
  ***Wing***. Light brown, veins dark brown, basicosta black; costal spines short and weak, shorter than r-m in length; dorsal and ventral surface of radial node all bare; distal parts of R4+5 and M straight; r-m and dm-cu not clouded; calypters and haltere brown-yellow.
  ***Legs***. All black; fore tibia with 2 median *p*, without long hairs on the ventral surface; mid femur with a row of short and weak *av*, a row of *pv* that are long and strong in the basal twothirds; mid tibia with 3–4 *p*, 1 *pv*, without *ad*; hind femur with *av*, short and weak *pv* in the apical half; hind tibia with 3 *av*, 3 *ad*, 1 *pd* in apical quarter, without sub-basal *pd*, apical *pv*; all tarsi longer than tibiae; claws and pulvilli short.
  ***Abdomen***. Black in ground color, nearly rounded in dorsal view, covered with gray pruinosity; each tergite with median black vitta, the posterior margins of tergites 3–5 with broad shifting stripes; sternite 1 bare.
  **Female. --** Unknown.
  **Type material. -- *Holotype***. China, Tibet, Motuo County, Mt. Duoxiongla, 3600–4200 m a.s.l., 8 August 2003, Mingfu Wang Collect., ♂ (IESNU). ***Paratype***. Same data as holotype. 2 ♂♂ (IESNU).
  **Remarks. --** This new species is similar to *P. latimargina* Fang *et* Fan, 1988, but it differs from the latter in the male: surfaces of upper ommatidia not enlarged; parafacial about 1.5 times as wide as the postpedicel; arista long plumose, the longest hair about 1.3 times as long as the width of the postpedicel; haustellum relatively shorter, about 1.5 times as long as the palpus at most; mid femur with a row of *pv* that are long and strong in the basal twothirds; mid tibia with 3–4 *p*, 1 *pv*; hind femur without *pv* in the basal half; hind tibia with 3 *ad*; abdomen nearly rounded in dorsal view.
  **Etymology. --** The species name is derived from the Latin words “*variatus*,” meaning shifting and “*marginis*,” meaning margin, referring to the posterior margins of tergites 3–5 having broad shifting stripes.
  **Distribution. --** China: Tibet.
**37. *P. wanfodinga*Feng *et* Ma, 2002: 98**
  **Distribution. --** China: Wanfoding (type loc.), Mt. Emei, Sichuan Province; Mt. Erlang, Yaan City, Sichuan Province.
**38. *P. wulinga*Xue, 1998: 1290**
  **Distribution. --** China: Mt. Leigong (type loc.), Leishan County, Guizhou Province.
**39. *P. zhangyeensis* Ma *et* Wu, 1992: 924**
  **Distribution. --** China: Zhangye City (type loc.), Gansu Province.
**40. *P. zhougongshana* Ma *et*Feng, 2002: 85**
  **Distribution. --** China: Mt. Zhougong (type loc.), Yaan City, Sichuan Province.

## Abbreviations:

***a***,: anterior seta;
***acr***,: acrostichal setae;
***ad***,: anterodorsal setae;
***av***,: anteroventral setae; collect., collector;
***dc***,: dorsocentral setae;
**dm-cu**,: medio-cubital cross-vein;
***fr***,: frontal setae;
***ial***,: intra-alar setae;
IESNU,: Institute of Entomology, Shenyang Normal University, China, loc., locality;
**M**,: medial vein.
***ors***,: orbital setae;
***p***,: posterior setae;
***pd***,: posterodorsal setae;
***post dc***,: postsutural dorsocentral setae;
***pra***,: prealar setae;
***prst acr***,: presutural acrostichal setae;
***prst dc***,: presutural dorsocentral setae;
***pv***,: posteroventral setae;
**R4+5**,: branch of radius;
**r-m**,: radio-medial cross-vein;
***v***,: ventral setae
